# The Effect of Drinking Ionized Water on the Productive Performance, Physiological Status, and Carcass Characteristics of Broiler Chicks

**DOI:** 10.3390/ani15020229

**Published:** 2025-01-16

**Authors:** Abdullah Mohamed, Mohamed Khalil, Farid Soliman, Karim El-Sabrout

**Affiliations:** Poultry Production Department, Faculty of Agriculture, Alexandria University, Alexandria 21545, Egypt; abdallah.hassan2@yahoo.com (A.M.); mohamedhassankhalil@yahoo.com (M.K.); nassif08@yahoo.com (F.S.)

**Keywords:** bacterial count, body weight, carcass quality, ORP, immunity, TAC, welfare

## Abstract

Water treatment technologies have received great attention recently, as water is the most important nutritional element, and animals consume it daily in larger quantities than those of food. The ideal water treatment affects the chemical composition and physical properties of water, having a significant positive impact on the animal’s physiology, productivity, and welfare. Studies conducted on water ionization devices for broiler chickens remain limited; therefore, this study was planned to investigate the effect of ionized drinking water on the productive performance, physiological status, and carcass characteristics of broiler chicks. A total of 900 one-day-old broiler chicks were randomly and equally assigned to three groups, each with six replicates (50 birds/replicate). The first group (C) received tap drinking water and served as a control, while the second group (T1) received ionized drinking water from an ionizing device that worked for 1 h/100 L. The third group (T2) received ionized drinking water from an ionizing device that worked for 2 h/100 L. Water analysis for each treatment was performed. Productive traits, such as weekly body weight, feed intake, and water intake, were recorded. Hematological parameters and biochemical constituents were measured according to the reference’s description. Furthermore, carcass characteristics, such as carcass weight and dressing percentage, and bacterial count of the intestine, such as Lactobacilli and Coliform counts, were determined. From the results, ionized water (T1 and T2) had a negative ORP, which is often desirable as it suggests the presence of antioxidant properties and lower total dissolved solids (TDSs), heterotrophic plate count (HPC), and algal total count (ATC) than in tap water. The treated chicks showed higher final body weights and better feed conversion rates than the control. Ionized water also improved carcass quality characteristics, such as carcass weight and dressing percentage. T1 and T2 chicks exhibited higher hemoglobin, total protein, globulin, G and M immunoglobulin, and total antioxidant capacity (TAC) levels, as well as lower malondialdehyde (MDA) and low-density lipoprotein (LDL) levels than the control. Furthermore, they had lower pathogenic bacteria counts. Therefore, it is recommended to employ the ionizing approach for broiler chicken drinking water, particularly a 2 h/100 L ionization application, for better animal productivity, health, and welfare.

## Simple Summary

Husbandry practices play a crucial role in maintaining the health and productivity of poultry birds, as well as in enhancing their well-being. Proper water quality management is one of the essential aspects of poultry husbandry, as water is a vital nutrient required for several biological functions in the bird’s body. Unfortunately, many poultry breeders provide their flocks with tap water that has unknown chemical, microbiological, and physical properties, particularly the oxidation–reduction potential (ORP), and impact on birds’ productivity and health. Therefore, it is imperative for broiler producers to monitor and maintain high water quality standards to ensure optimal health and performance in their flocks. The ionization process can enhance drinking water quality and improve the health and productivity of broiler chicks.

## 1. Introduction

Large-scale husbandry approaches are a major contributor to reducing animal stress, environmental footprint, and public health threats. Water treatment technologies have received great attention recently, as water is the most important nutritional element for living organisms. Water quality is essential for the proper growth and development of broiler chicks. It is well known that poor water quality can lead to welfare issues in poultry. As the poultry industry continues to grow and evolve, it is essential for farmers to continue exploring innovative water treatment methods like ionization to ensure the health and well-being of their flocks. Water ionization is a process that involves the use of positively and negatively charged ions to remove impurities from water. This method is particularly effective in killing harmful microorganisms that may be present in the water supply; so, it has gained popularity in recent years [1,2]. Accordingly, broiler chicks can be provided with clean and safe drinking water, which can lead to healthier and more productive birds. Furthermore, ionization has been found to promote the overall quality of water for broiler chicks [3]. By removing contaminants such as chlorine and heavy metals like cadmium, ionization can create a more balanced pH level in the water [4]. This is important for maintaining the health of broiler chicks, as fluctuations in pH can lead to digestive issues and other health problems.

Ionized water typically refers to water that has been electrolyzed, resulting in a slightly alkaline solution [1,3]. Generally, the electrostatic treatment of water is gaining great attention as a novel, multifunctional disinfection practice and health enhancer. It is a valuable tool for poultry breeders looking to improve the quality of water for their flocks. The treated water is suggested to affect the physiological and productive traits of birds by contributing to increased molecular ionization and fluidity, which improves several biological activities [3,5,6]. Working on various types of drinking water (ionized water, magnetic water, and tap water), Jassim and Aqeel [3] revealed that ionized alkaline water has a positive effect on some biochemical parameters in broiler chickens. Alkaline drinking water also promotes broiler growth performance, immunity, and digestive physiology [7]. In the same manner, Shihab et al. [4] found that using ionized alkaline drinking water has beneficial effects on the physiological and growth performances of Japanese quails. Moreover, alkaline electrolyzed water can reduce the number of aerobic bacteria in chicken carcasses while delaying chemical alterations, increasing the shelf life of chicken meat, and improving its overall odor, texture, and color [2].

The previous studies that have addressed broiler drinking water have focused on the water pH (acidity and alkalinity) effects on birds’ performance without considering the oxidation–reduction potential (ORP) impact. The water ORP is a parameter to measure the capacity of a sanitizer to be a strong oxidizer for destroying microorganisms or for reacting with some harmful minerals, such as chlorine and iron [8]. However, the results of these previous studies are also not consistent due to several factors, such as differences in the water source, the device type, the surrounding environment, and the bird’s overall health and species. Hence, the current study investigated the effect of ionized drinking water, focusing on the ORP factor influence, on the productive performance, hematological parameters, blood biochemical indices, immunological response, intestinal bacterial count, internal organs, and carcass characteristics of broiler chicks.

## 2. Materials and Methods

### 2.1. Animal Ethics

All experimental procedures were conducted in accordance with the ethical guidelines established by the Animal Care Committee of Alexandria University (Alex. Agri. 092406132).

### 2.2. Experimental Design

**Figure 1:**
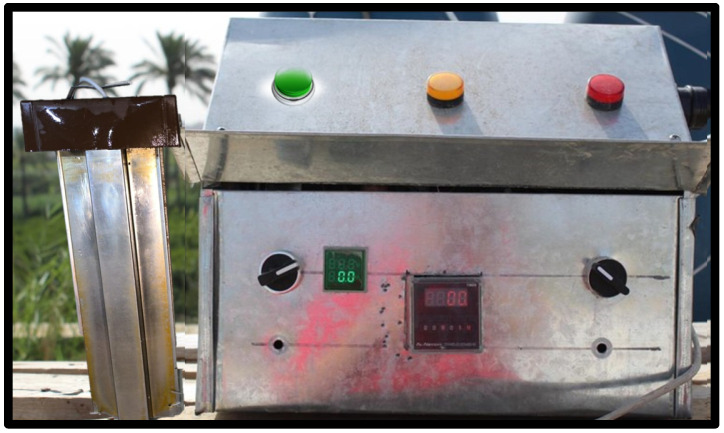
The water ionizing device.

The experiment was conducted in the spring (October–November) of 2024 at a private broiler farm in Alexandria (31.2001° N, 29.9187° E). A total of 900 one-day-old Cobb 500 broiler chicks, with a similar initial body weight of approximately 36.5 g, were randomly and equally assigned to 3 groups. Each group consisted of 6 replicates (50 birds/replicate), with each replicate housed in a separate pen. The 1st group (C) received tap drinking water and served as a control, while the 2nd group (T1) received ionized drinking water (the ionizing device worked for 1 h/100 L). The 3rd group (T2) also received ionized drinking water (the ionizing device worked for 2 h/100 L). Ionized water was produced electrically using a local device of Al-Nessim Company (WID 12 V—30,000 Hz, Cairo, Egypt; Figure 1), by passing electric ions through an electrode placed in a water tank (100 L) for certain periods (1 h and 2 h). The experiment lasted for 37 days, corresponding to the slaughter age of the chicks.

### 2.3. Animal Husbandry

In an open-system building, the broiler chicks were housed on wood shaving bedding. They were raised under similar environmental conditions and hygienic management. Gas heaters were used to provide the birds with the needed heat for brooding; room temperature was about 33 °C for the first three days and then decreased by 0.3 °C daily until reaching 23 °C. The chicks were subjected to a continuous illumination program consisting of 23 h of light and 1 h of darkness. They were given ad libitum access to a commercial diet “starter” containing 23% crude protein (CP) and 2950 kcal/kg of metabolizable energy (ME) during the brooding stage (1–21 days), while they were fed a grower–finisher diet containing 21% CP and 3150 kcal/kg ME during the fattening stage (22–37 days), along with fresh clean water.

### 2.4. Data Collection

#### 2.4.1. Water Analysis

The physical, chemical, and microbiological properties of water were determined using a pH meter and a multifunction water meter (DULVAN 7 in 1 Tester Pen, Beijing, China), as well as by performing phytoplankton examination and plate count determination.

#### 2.4.2. Productive Performance

The birds were individually weighed on day 1. The body weight, body weight gain, feed intake, feed conversion rate, water consumption, and mortality rate of the chicks were recorded weekly during the experiment.

#### 2.4.3. Hematological and Biochemical Parameters

Following a 37-day experimental period, a random selection of 10 chicks from each group was made. Blood samples were collected from these chicks during slaughter for subsequent hematological and biochemical examinations. Red blood cell (RBC) counts were determined using an acridine orange (AO) bright-line hemocytometer under a light microscope at 400× magnification. White blood cell (WBC) counts were determined according to the methods described by El-Saadany et al. [9] and El-Prollosy et al. [10]. Hematocrit percentages were estimated using Wintrobe hematocrit tubes, while hemoglobin (Hb) concentration was measured using the cyanmethemoglobin method. Total protein and albumin concentrations were measured as described by the manufacturer, while globulin concentration was calculated by subtracting the albumin value from the total protein value. Liver enzymes’ activity (aspartate aminotransferase (AST) and alanine aminotransferase (ALT)) was assayed in plasma by the method of Reitman and Frankel [11] using a specific kit (Diamond Diagnostics Chemical Company, Cairo, Egypt). Creatinine and uric acid were evaluated to determine kidney function. Plasma glucose concentration was determined using the instructions provided with a specific kit (Diamond Diagnostic Company, Egypt) and the method described by Trinder [12]. Plasma triiodothyronine (T_3_) levels were quantified using radioimmunoassay (RIA) kits (Diagnostic Systems Laboratories, Webster, TX, USA) according to the method of Hollander and Shenkman [13]. Additionally, the plasma immunoglobulin A (IgA) and G (IgG) concentrations were determined using chicken ELISA kits (enzyme-linked immunosorbent assay kit, Cloud-Clone Corp., Katy, TX, USA), while immunoglobulin M (IgM) was assessed using an IgM ELISA kit (Immunology Consultants Laboratory, Inc., Portland, OR, USA), according to the manufacturer’s instructions. Plasma total antioxidant capacity (TAC) and malondialdehyde (MDA) were determined according to Benzie and Strain [14] and Placer et al. [15], respectively. According to Lacková et al. [16], a lipid profile assessment was performed, which included measurements of total cholesterol (TC), high-density lipoprotein (HDL) cholesterol, low-density lipoprotein (LDL) cholesterol, and triglycerides (TGs).

#### 2.4.4. Carcass Quality and Internal Organs

At the end of the experiment, the chickens were slaughtered three hours after feed withdrawal. Ten birds from each group were randomly chosen to evaluate carcass quality parameters, including carcass weight and dressing percentage. Additionally, small intestine length and internal organ relative weights were measured. The birds were weighed and slaughtered by cervical dislocation, followed by exsanguination. After the removal of feathers, viscera, shanks, neck, and head, the individual weights of the eviscerated hot carcass and the relative weight of the internal organs (expressed as percentages) were measured for each bird. Additionally, the breast muscle’s pH was measured 30 min after slaughter using a digital pH meter (D-MultiTester Pen, Beijing, China). The breast color was determined after slaughter by Hunter Lab ColorFlex EZ, Reston, VA, USA. The values of L*, a*, and b* indicate lightness, redness, and yellowness, respectively.

#### 2.4.5. Intestinal Microbiota

Intestinal bacterial samples were collected from 10 chickens per group at the end of the experiment (after slaughtering) in sterile tubes and stored at 2 °C until microbial analysis. *Lactobacillus plantarum*, *Salmonella enteritidis*, *Coliform* spp., and *Escherichia coli* were counted as described by Donato et al. [17]. The collected intestinal content homogenates were serially diluted from 10^−1^ to 10^−7^, and selected agar media were employed to enumerate the targeted bacterial groups. The culture plates were then incubated at 37 °C for 48 h. A colony counter was used for the enumeration of visible colonies, and the results are expressed as log_10_ CFU/g of cecal content.

#### 2.4.6. Statistical Analysis

Data were analyzed through one-way analysis of variance (ANOVA) in a completely randomized design (CRD) using the general linear model (GLM) procedure in accordance with the SAS program (Version 15.1 2018, USA). All values are presented as means with a standard error of the mean. Significant differences among treatments were subjected to Tukey’s test. The results were considered significant at *p* ≤ 0.05.

## 3. Results

Table 1 shows the physical, chemical, and microbiological characteristics of drinking water for broilers. Significant differences (*p* ≤ 0.05) were detected among the three groups. The treated water (T1 and T2) showed lower levels of total dissolved solids (TDSs), heterotrophic plate count (HPC), and algal total count (ATC) than the control. Ionized water also exhibited higher Na and Cl levels than tap water. T2 (ionization for 2 h) had the highest pH and the least ORP values. There were no significant (*p* > 0.05) differences in electrical conductivity (EC), salinity (%), and *E. coli* count among the groups.

From the results of Table 2, ionized drinking water positively (*p* ≤ 0.05) affected the chicks’ productive performance. The ionized water groups (T1 and T2) had higher water consumption (12.35%) and lower mortality rate (58.47%) than the control group. T2 chicks exhibited the highest final body weight (FBW) (*p* = 0.001) and body weight gain (BWG) (*p* = 0.002), the best feed conversion rate (FCR) (*p* = 0.037), and the lowest mortality percentage (*p* = 0.004). There were no significant (*p* > 0.05) differences in feed intake among the groups.

According to the results of Table 3, ionized drinking water impacted broiler chicks’ hematological and blood biochemical parameters. In comparison to the control, T1 and T2 chicks had higher (*p* ≤ 0.05) Hb and hematocrit levels (within the normal range), while there were no significant (*p* > 0.05) differences in RBC and WBC values among the groups. Chicks in T1 and T2 had significantly (*p* ≤ 0.05) higher total protein (TP), globulin (Glb), IgG, IgM, TAC, and T_3_ levels than the control. They also had significantly (*p* ≤ 0.05) lower glucose, total cholesterol, LDL, and MDA levels. There were no significant (*p* > 0.05) differences in the AST, ALT, creatinine, and uric acid values among the groups.

Table 4 presents the effect of ionized drinking water on carcass characteristics and the relative weight of the internal organs of the chicks. From the results, T1 and T2 chicks had higher (*p* ≤ 0.05) carcass weight, dressing percentage, and intestinal length compared to the control. T2 chicks had the highest carcass weight (13.67%), while there were no significant (*p* > 0.05) differences in carcass pH, breast color, and heart, liver, gizzard, and spleen percentage relative weights among the studied groups.

Based on the data presented in Table 5, ionized drinking water had a significant (*p* ≤ 0.05) impact on the intestinal bacterial counts of broiler chicks. T1 and T2 chicks had higher counts (*p* ≤ 0.05) of *Lactobacillus* spp. and lower counts of (*p* ≤ 0.05) *Coliform* sp. and *E. coli* than the control.

## 4. Discussion

Providing enriched husbandry such as access to high-quality drinking water is crucial for maintaining optimal hydration levels, regulating body temperature, aiding digestion, reducing stress, and ultimately supporting overall physical health and productivity in animals [18,19,20]. Therefore, it has become necessary to develop water treatment methods able to remove pollutants that are still left behind by traditional methods and some chemicals that contribute to the loss of the vital properties of water and cause various health problems, as low water quality represents one of the main challenges facing poultry farming [21]. Therefore, the overall tendency is toward ionized water, which is functional water with noticeable features. There are many specialized ways to produce it, such as electrolysis, which requires passing electrical ions between a positive electrode and a negative electrode inside water, resulting in healthy, beneficial drinking water with a negative ORP that has antioxidant properties and a lower bacterial and algae content than tap water [22]. Additionally, electrolyzing drinking water enriches water quality by increasing molecular ionization and fluidity, which improves birds’ biological activities [1,2,3,4,5].

According to the results of the water analysis shown in Table 1, the ionization process of broiler chicken drinking water positively affected water quality parameters, including ORP, pH, TDSs, and ATC. The oxidation–reduction potential (ORP) is a measurement of ion exchange. Substances with a negative ORP value can donate extra ions, but positive ORP values lead to ion absorption. Water with a positive ORP can be harmful, similar to water with a low pH [8,22,23]. So, ORP and pH level are generally taken care of in processed and treated water. Otherwise, when your water has a negative ORP value, it has antioxidant properties. This explains the superior effect of ionized water, particularly for T2, on broilers’ performance in the present study. This is also associated with cellular health, as a negative ORP value in drinking water can promote electron release to neutralize free radicals. Free radicals are unstable ions that are generated by the body and can cause undesirable reactions with proteins, lipids, and DNA [24,25]. However, the increased production of reactive oxygen species (ROS) and free radicals results in oxidative stress, which alters animal overall performance [25]. Hence, drinking water having antioxidant properties, such as ionized water, scavenges ROS and is beneficial in decreasing MDA (a radical oxidative marker) levels and mitigating oxidative stress. In agreement with this, Ezzat et al. [1] discovered that treated alkaline drinking water can reduce oxidative stress, which is considered a major concern that impacts the overall health of chickens in modern production systems, especially in tropical and subtropical regions. Additionally, ORP is a measurement of the cleanliness of water and its ability to break down contaminants. Water’s ORP level has a significant effect in killing several pathogenic microorganisms [23,26]. In this sense, a negative ORP can be seen as an indicator of water’s ability to purify itself and eliminate harmful substances. While a negative ORP can provide some insight into the cleanliness and potential health benefits of water, it should not be treated as the sole indicator of water safety. By considering a range of factors in water quality assessment, we can make informed decisions about our drinking water and protect our health. However, the ORP values are affected by several factors, including pH [27]. Physically, the exposure of water to an electric field changes the water’s properties, including raising the pH, TAC, and the levels of vital minerals, such as Ca and Na, while reducing TDSs, HPC, and ATC, as presented in the current study (Table 1). Moreover, treating drinking water might cause the hydrogen–oxygen bond angle within the water molecule to be reduced in degrees [28]. This causes the water molecules to cluster together in groups that are smaller than those found in ordinary water. These smaller clusters lead to better absorption of water into the cell.

However, the results of this study are promising, as ionized drinking water showed several advantages, including better marketing body weight (MBW), FCR, Hb, TP, Glb, T_3_, IgM, and IgG levels, TAC, and dressing percentages, in addition to reduced MDA, LDL, harmful intestinal bacteria (e.g., *E. coli*), and mortality percentages (Table 2, Table 3, Table 4 and Table 5). Therefore, this approach can be widely applied in broiler farms as a low-cost, ecologically friendly, and safe tool to enhance birds’ health, welfare, and production. Tap water has a pH of around 7, whereas ionized water in the current study reached a pH of 7.6. Slightly alkaline drinking water has been shown to improve the productive, physiological, and immunological traits of certain poultry species [1,29]. Hence, alkaline water could be recommended as the main drinking water source for broiler chickens, as it improves the growth performance, carcass characteristics, meat quality, and immune status, as well as decreases the mortality rates of birds [30,31]. Ionized water, particularly alkaline ionized water, may influence the metabolic processes in the bird, leading to altered pH levels in the muscle tissues. It is also rich in antioxidants, which can help neutralize free radicals and reduce oxidative stress, potentially influencing carcass and meat pH. The treated alkaline water has excessive oxygen in the form of OH− instead of O_2_, which is more stable and inhibits the formation of scale [5,32]. Proper hydration is essential for supporting metabolic processes, regulating body temperature, and promoting overall well-being in poultry. In this sense, El-Sabrout and Hanafy [5] indicated that water with an alkaline pH can be recommended in poultry production, as it helps the birds reduce stress and leads to better performance [33]. According to [34,35], an optimal balance of electrolytes in drinking water for broilers regulates blood and fluid retention. In this study, we observed that the treated chick groups exhibited higher water consumption than the control. This observation could be due to the good taste of ionized drinking water and its higher sodium level compared to the control water (tap water). Furthermore, drinking water with lower levels of TDSs can increase palatability and water consumption, as mentioned by Abbas et al. [36]. However, the drinking water supplied to broilers should not contain excessive levels of TDSs, as they can decrease the productive performance and increase the mortality rate [37].

Recently, several studies have addressed drinking water quality management for poultry. In agreement with the current research findings, Ebrahimi et al. [20] emphasized the need to implement water treatment and quality improvement measures for poultry drinking water, as the physicochemical composition of water can affect the amount of water consumed and performance in birds. Khan et al. [38] indicated that water from different sources could vary in the degree of acidity (pH), and birds that drank slightly alkaline water (~7.5) showed highly significant gains in body weight and feed conversion efficacy compared to the control group (receiving ordinary water with an apparently neutral pH). Similarly, Shihab et al. [4] revealed that the productive and physiological performance of Japanese quail was improved using alkaline drinking water. Blood biochemical/hematological and internal organ measurements monitor physiological activity changes and reflect animal health and production [39]. In the current experiment, the Hb, TP, Glb, and T_3_ levels were increased in the treated chick groups, while the blood total cholesterol and LDL levels were decreased. Ezzat et al. [40] demonstrated that alkaline ionized water has beneficial effects on quail physiological performance, as it increases some blood indices like Hb level and PCV. Moreover, they revealed that villus height, crypt depth, and mucous layer thickness in the duodenum were increased in the treated birds. However, we found a significant increase in intestinal length (7.63%) compared to the control, which has a substantial effect on nutrient absorption and in increasing growth performance. Jassim and Aqeel [3] observed productive performance improvement as a result of using alkaline drinking water to enhance certain blood biochemical traits of birds. The slight increase in some blood hormones involved in growth, such as T_3_, may also have a direct reflection in birds’ performance improvement. Mahbuba [41] explained this improvement as promoting the thyroid gland’s action in the release of the thyroxin hormone, which results in increased feed consumption, as well as improved fat and protein metabolism. Additionally, treated drinking water can help in the re-growth and renewal of cells and increases the passing of ions through cell membranes [42]. Based on the results of Table 4, there were no significant differences in heart, liver, gizzard, and spleen percentage relative weights among the studied groups, but there were significant increases in carcass weight and dressing percentages for the treated chicks. These positive outcomes can be attributed to improvements in the blood biochemical and hematological parameters, alongside the enhanced overall performance and welfare of the chicks. Notably, the observed increase in intestinal length, potentially a consequence of enriched water stimulation, significantly contributed to improved water intake, feed conversion efficiency, and ultimately, higher final body weights [39]. Nevertheless, highly alkalized water is not desirable, as mentioned by Martínez et al. [7]. Alkalizing drinking water with a high amount of NaHCO_3_ changed the normal parameters of drinking water for broilers, and this treatment also provoked poor productive development, high frequency of ascites, and mortality, besides adversely modifying the relative weight of the immune organs, such as the spleen, as well as the cecal pH and bacterial count. Therefore, the supply of alkaline water should be carefully considered because it has a direct impact on bird’s health and productivity and the economy of the broiler producer.

One of the key factors affecting water quality in poultry husbandry is the presence of contaminants, such as bacteria, algae, fungi, and chemicals. These contaminants can enter the water supply through various sources, including contaminated equipment and the surrounding environment. Regular testing of water quality parameters, including TDS and microbial content, is essential for identifying and addressing potential issues that may impact poultry health and performance. Implementing appropriate water treatment protocols, such as ionization, can help mitigate the risk of waterborne diseases and ensure that birds have access to clean and safe drinking water. Exposing water to electric or magnetic fields affects the water components, including the mineral content and microbial count, and the effects depend upon the strength of the electric/magnetic field and the exposure time. From this perspective, the most significant advantage of using ionized water to inactivate bacteria is the lack of the need of chemicals [43]. According to the results of Table 5, the use of ionized alkaline water given to chicks had a beneficial influence on their intestinal health by enhancing gut integrity and modulating the gut microflora by reducing the colonization of intestinal pathogenic bacteria and promoting competitive exclusion. In addition, this treated water is an efficient and safe disinfectant due to its anti-bacterial properties [2,8]. In the current study, ionized drinking water increased the counts of beneficial bacteria (Lactobacilli) and decreased those of harmful bacteria (Coliform and *E. coli*). These results are consistent with those of Ezzat et al. [40], who observed that ionized drinking water increased the number of Lactobacilli bacteria and decreased the number of Coliform bacteria in quails. Furthermore, ionizing water in our study, particularly for 2 h, slightly raised the level of chlorine, which has the most profound effect as a disinfectant against pathogenic bacteria, as reported by [2,44].

Based on these findings, ionized water, particularly alkaline ionized water, can help maintain a more optimal pH balance in the bird’s body, which can have a positive impact on various physiological processes, including digestion, nutrient absorption, and muscle metabolism. Ionized alkaline water may alter the molecular structure of water, making it more easily absorbed by the intestinal cells of birds. This improved absorption can lead to a better utilization of nutrients from feed, potentially resulting in higher growth rates and body weights. Additionally, a more efficient nutrient utilization can lead to a lower feed conversion ratio, reducing the feed costs. Furthermore, ionized water has antioxidant and anti-inflammatory properties, which could help reduce the stress levels, strengthen the immune system of the birds, lower their mortality rates, and enhance carcass quality. Therefore, by ionizing tap water, as a green technology, broiler breeders can guarantee that their birds are receiving water that fulfills their specific needs for optimal growth and better health without an adverse impact on the overall physiological status. However, further studies on employing higher ionization levels for better chick husbandry are required.

## 5. Conclusions

Successful water enrichment strategies can provide direct and indirect benefits for broiler chicks’ health, welfare, and productivity. Broiler farmers can safely use ionization as a water treatment technology to ensure that their birds receive water that fulfills their individual needs for optimal growth and health. According to the current findings, ionizing drinking water, particularly for 2 h/100 L, positively affected broiler chickens’ productive and physiological traits, as well as enhanced immunity and carcass quality. Therefore, it is recommended to apply such treatment tools to drinking water in broiler farms as a low-cost approach to improve chickens’ overall health and general production parameters.

## Figures and Tables

**Table 1:** Analyses of drinking tap and ionized water used in this study for broiler chickens.

Parameters	C (Control)	T1 (Ionized Water 1 h)	T2 (Ionized Water 2 h)	SEM	*p*-Value
pH	7.11 ^c^	7.26 ^b^	7.35 ^a^	±0.01	0.038
ORP (mV)	680 ^a^	−200 ^b^	−400 ^c^	±0.02	0.001
Salinity (%)	0.03	0.03	0.03	±0.00	0.188
TDSs (ppm)	357 ^a^	345 ^b^	339 ^c^	±0.03	0.041
EC (µs/cm)	713	697	692	±0.02	0.061
ATC (unit/mL)	57 ^a^	18 ^b^	13 ^c^	±0.01	0.001
HPC (Cfu/1 mL)	3 ^a^	<1 ^b^	<1 ^b^	±0.00	0.001
*E. coli* (Cfu/100 mL)	<1	<1	<1	±0.00	0.198
Na^+^ (meq/L)	2.9 ^b^	3.3 ^a^	3.5 ^a^	±0.01	0.045
K^+^ (meq/L)	0.3	0.3	0.3	±0.00	0.540
Ca^2+^ (meq/L)	1.1 ^b^	1.4 ^a^	1.6 ^a^	±0.00	0.021
Mg^2+^ (meq/L)	0.8	0.8	0.8	±0.00	0.612
Cl^–^ (meq/L)	2.4 ^b^	2.8 ^a^	3.0 ^a^	±0.01	0.048
HCO_3_^−^ (meq/L)	2.0	2.1	2.2	±0.00	0.058
CO_3_^2−^ (meq/L)	0.0	0.0	0.0	±0.00	0.718
SO_4_^2−^ (meq/L)	1.1	1	0.9	±0.00	0.065

^a,b,c^ Means having different letters in the same row are significantly different (*p* ≤ 0.05).

**Table 2:** The effect of ionized drinking water on the productive performance of broiler chicks.

	Treatments	C (Control)	T1 (Ionized Water 1 h)	T2 (Ionized Water 2 h)	*p*-Value
Traits					
FBW (g)		2095.22 ^c^ ± 6.55	2162.45 ^b^ ± 7.01	2305.31 ^a^ ± 6.60	0.001
BWG (g)		2058.17 ^c^ ± 5.84	2125.24 ^b^ ± 5.89	2268.13 ^a^ ± 5.83	0.002
FI (g)		3155.15 ± 1.18	3168.45 ± 1.10	3173.02 ± 1.14	0.060
FCR		1.58 ^a^ ± 1.15	1.44 ^b^ ± 1.13	1.39 ^c^ ± 1.15	0.037
WC (L)		5.71 ^b^ ± 1.05	6.34 ^a^ ± 1.03	6.49 ^a^ ± 1.05	0.025
Mortality (%)		4.19 ^a^ ± 0.13	2.03 ^b^ ± 0.11	1.46 ^c^ ± 0.10	0.004

^a,b,c^ Means having different letters in the same row are significantly different (*p* ≤ 0.05). FBW = final body weight at 37 days of age. BWG = body weight gain from 1 to 37 days. FI = feed intake from 1 to 37 days. FCR = feed conversion rate. WC = total water consumption.

**Table 3:** The effect of ionized drinking water on the blood hematological and biochemical parameters of broiler chicks.

	Treatments	C (Control)	T1 (Ionized Water 1 h)	T2 (Ionized Water 2 h)	*p*-Value
Parameters					
RBC_S_ (10^6^/mm^3^)		2.53 ± 0.09	2.69 ± 0.05	2.75 ± 0.04	0.061
Hb (g/dL)		9.95 ^b^ ± 0.51	10.92 ^a^ ± 0.73	11.18 ^a^ ± 0.60	0.045
Hematocrit (%)		29.41 ^c^ ± 0.63	32.02 ^b^ ± 0.55	33.85 ^a^ ± 0.62	0.041
WBC_S_ (10^3^/mm^3^)		19.88 ± 0.53	20.36 ± 0.54	20.55 ± 0.51	0.068
Total protein (g/dL)		3.52 ^b^ ± 0.12	4.73 ^a^ ± 0.09	4.85 ^a^ ± 0.14	0.031
Albumin (g/dL)		2.15 ^b^ ± 0.04	2.52 ^a^ ± 0.06	2.63 ^a^ ± 0.06	0.030
Globulin (g/dL)		1.37 ^b^ ± 0.05	2.19 ^a^ ± 0.05	2.21 ^a^ ± 0.06	0.028
Glucose (mg/dL)		185.11 ^a^ ± 11.05	171.11 ^b^ ± 11.08	172.80 ^b^ ± 12.01	0.045
Triglycerides (mg/dL)		136.60 ± 2.28	131.25 ± 2.35	129.13 ± 2.22	0.063
Cholesterol (mg/dL)		167.20 ^a^ ± 10.13	121.12 ^b^ ± 11.08	118.44 ^b^ ± 11.01	0.015
HDL (mg/dL)		45.44 ± 0.60	45.72 ± 0.57	46.03 ± 0.51	0.062
LDL (mg/dL)		62.02 ^a^ ± 11.03	51.27 ^b^ ± 12.01	49.89 ^b^ ± 12.13	0.033
AST (U/L)		142.91 ± 5.60	143.80 ± 6.15	147.60 ± 6.20	0.072
ALT (U/L)		24.01 ± 1.39	22.80 ± 1.52	21.97 ± 1.58	0.063
Creatinine (mg/dL)		0.68 ± 0.02	0.71 ± 0.04	0.72 ± 0.04	0.060
Uric acid (mg/dL)		2.23 ± 0.03	2.28 ± 0.03	2.30 ± 0.04	0.058
IgA (mg/mL)		0.96 ± 0.01	0.97 ± 0.01	1.01 ± 0.02	0.124
IgG (mg/mL)		1.13 ^b^ ± 0.18	1.45 ^a^ ± 0.18	1.44 ^a^ ± 0.17	0.048
IgM (mg/mL)		0.22 ^b^ ± 0.20	0.38 ^a^ ± 0.19	0.41 ^a^ ± 0.20	0.006
T_3_ (ng/dL)		2.95 ^b^ ± 0.08	3.84 ^a^ ± 0.09	3.91 ^a^ ± 0.08	0.001
TAC (mmol/L)		1.14 ^b^ ± 0.03	1.72 ^a^ ± 0.05	1.85 ^a^ ± 0.03	0.002
MDA (nmol/mL)		4.13 ^a^ ± 0.05	2.95 ^b^ ± 0.05	2.79 ^b^ ± 0.06	0.005

^a,b,c^ Means having different letters in the same row are significantly different (*p* ≤ 0.05). RBCs = red blood cells. Hb = hemoglobin. WBC_S_ = white blood cells. HDL = high-density lipoprotein. LDL = low-density lipoprotein. AST = aspartate aminotransferase. ALT = alanine transaminase. IgA = immunoglobulin A. IgG = immunoglobulin G. IgM = immunoglobulin M. T_3_ = triiodothyronine. TAC = total antioxidant capacity. MDA = malondialdehyde.

**Table 4:** The effect of ionized drinking water on carcass characteristics and the relative weight of internal organs in broiler chicks.

	Treatments	C (Control)	T1 (Ionized Water 1 h)	T2 (Ionized Water 2 h)	*p*-Value
Characteristics					
Carcass weight (g)		1537.04 ^c^ ± 5.30	1619.45 ^b^ ± 5.21	1747.18 ^a^ ± 5.32	0.002
Lightness (L*)		51.08 ± 0.28	52.01 ± 0.32	51.97 ± 0.30	0.085
Redness (a*)		0.66 ± 0.35	0.69 ± 0.32	0.67 ± 0.33	0.079
Yellowness (b*)		10.75 ± 0.27	11.05 ± 0.28	10.81 ± 0.27	0.068
Breast pH		6.21 ± 0.11	6.05 ± 0.12	5.88 ± 0.12	0.064
Dressing (%)		73.29 ^c^ ± 0.51	74.85 ^b^ ± 0.49	75.78 ^a^ ± 0.40	0.039
Heart (%)		0.39 ± 0.10	0.40 ± 0.11	0.41 ± 0.11	0.071
Liver (%)		2.43 ± 0.02	2.46 ± 0.01	2.47 ± 0.02	0.125
Gizzard (%)		1.57 ± 0.02	1.64 ± 0.02	1.65 ± 0.02	0.078
Spleen (%)		0.13 ± 0.01	0.15 ± 0.02	0.16 ± 0.01	0.062
Intestine length (cm)		177.03 ^c^ ± 3.98	185.19 ^b^ ± 4.11	192.01 ^a^ ± 4.13	0.005

^a,b,c^ Means having different letters in the same row are significantly different (*p* ≤ 0.05).

**Table 5:** The effect of ionized drinking water on the intestinal bacterial count of broiler chicks.

	Treatments	C (Control)	T1 (Ionized Water 1 h)	T2 (Ionized Water 2 h)	*p*-Value
Bacteria Species					
*Lactobacillus* spp. (log_10_ CFU/g)		6.40 ^b^ ± 0.14	7.12 ^a^ ± 0.11	7.18 ^a^ ± 0.12	0.031
*Salmonella enteritidis* (log_10_ CFU/g)		0.70 ± 0.16	0.69 ± 0.15	0.66 ± 0.15	0.059
*Coliform* ssp. (log_10_ CFU/g)		3.89 ^a^ ± 0.12	3.34 ^b^ ± 0.11	3.31 ^b^ ± 0.11	0.022
*Escherichia coli* (log_10_ CFU/g)		6.01 ^a^ ± 0.13	5.02 ^b^ ± 0.13	4.93 ^b^ ± 0.13	0.031

^a,b^ Means having different letters in the same row are significantly different (*p* ≤ 0.05).

## Data Availability

The supplementary data can be available from the corresponding author upon a reasonable request.
