# Correction: Mohamed et al. The Effect of Drinking Ionized Water on the Productive Performance, Physiological Status, and Carcass Characteristics of Broiler Chicks. *Animals* 2025, *15*, 229

**DOI:** 10.3390/ani15213133

**Published:** 2025-10-29

**Authors:** Abdullah Mohamed, Mohamed Khalil, Farid Soliman, Karim El-Sabrout

**Affiliations:** Poultry Production Department, Faculty of Agriculture, Alexandria University, Alexandria 21545, Egypt; abdallah.hassan2@yahoo.com (A.M.); mohamedhassankhalil@yahoo.com (M.K.);

## Text Correction

There was a mistype in the original publication [1] due to authors’ oversight. A correction has been made to Section 2. Materials and Methods, 2.3 Animal Husbandry, Paragraph 1:

Gas heaters were used to provide the birds with the needed heat for brooding; room temperature was about 33 °C for the first three days and then decreased by 0.3 °C daily until reaching 23 °C.

There was an error in the original publication [1] due to authors’ oversight. A correction has been made to Section 2. Materials and Methods, 2.4 Data Collection, 2.4.5 Intestinal Microbiota, Paragraph 1:

Intestinal bacterial samples were collected from 10 chickens per group at the end of the experiment (after slaughtering) in sterile tubes and stored at 2 °C until microbial analysis.

## Table Correction

In the original publication [1], there was a mistake in Table 3 as published. Creatinine (mg/dL) values of T1 and T2 were shifted with their standard errors, most likely, during the conversion of the DOCX file to PDF. The corrected Table 3 appears below.

The authors would like to apologize for any inconvenience to the readers caused by these unintentional errors. They also state that the scientific results and conclusions are unaffected. This correction was approved by the Academic Editor. The original publication has also been updated.

## Figures and Tables

**Table 3:** The effect of ionized drinking water on the blood hematological and biochemical parameters of broiler chicks.

	Treatments	C (Control)	T1 (Ionized Water 1 h)	T2 (Ionized Water 2 h)	*p*-Value
Parameters					
RBC_S_ (10^6^/mm^3^)		2.53 ± 0.09	2.69 ± 0.05	2.75 ± 0.04	0.061
Hb (g/dL)		9.95 ^b^ ± 0.51	10.92 ^a^ ± 0.73	11.18 ^a^ ± 0.60	0.045
Hematocrit (%)		29.41 ^c^ ± 0.63	32.02 ^b^ ± 0.55	33.85 ^a^ ± 0.62	0.041
WBC_S_ (10^3^/mm^3^)		19.88 ± 0.53	20.36 ± 0.54	20.55 ± 0.51	0.068
Total protein (g/dL)		3.52 ^b^ ± 0.12	4.73 ^a^ ± 0.09	4.85 ^a^ ± 0.14	0.031
Albumin (g/dL)		2.15 ^b^ ± 0.04	2.52 ^a^ ± 0.06	2.63 ^a^ ± 0.06	0.030
Globulin (g/dL)		1.37 ^b^ ± 0.05	2.19 ^a^ ± 0.05	2.21 ^a^ ± 0.06	0.028
Glucose (mg/dL)		185.11 ^a^ ± 11.05	171.11 ^b^ ± 11.08	172.80 ^b^ ± 12.01	0.045
Triglycerides (mg/dL)		136.60 ± 2.28	131.25 ± 2.35	129.13 ± 2.22	0.063
Cholesterol (mg/dL)		167.20 ^a^ ± 10.13	121.12 ^b^ ± 11.08	118.44 ^b^ ± 11.01	0.015
HDL (mg/dL)		45.44 ± 0.60	45.72 ± 0.57	46.03 ± 0.51	0.062
LDL (mg/dL)		62.02 ^a^ ± 11.03	51.27 ^b^ ± 12.01	49.89 ^b^ ± 12.13	0.033
AST (U/L)		142.91 ± 5.60	143.80 ± 6.15	147.60 ± 6.20	0.072
ALT (U/L)		24.01 ± 1.39	22.80 ± 1.52	21.97 ± 1.58	0.063
Creatinine (mg/dL)		0.68 ± 0.02	0.71 ± 0.04	0.72 ± 0.04	0.060
Uric acid (mg/dL)		2.23 ± 0.03	2.28 ± 0.03	2.30 ± 0.04	0.058
IgA (mg/mL)		0.96 ± 0.01	0.97 ± 0.01	1.01 ± 0.02	0.124
IgG (mg/mL)		1.13 ^b^ ± 0.18	1.45 ^a^ ± 0.18	1.44 ^a^ ± 0.17	0.048
IgM (mg/mL)		0.22 ^b^ ± 0.20	0.38 ^a^ ± 0.19	0.41 ^a^ ± 0.20	0.006
T_3_ (ng/dL)		2.95 ^b^ ± 0.08	3.84 ^a^ ± 0.09	3.91 ^a^ ± 0.08	0.001
TAC (mmol/L)		1.14 ^b^ ± 0.03	1.72 ^a^ ± 0.05	1.85 ^a^ ± 0.03	0.002
MDA (nmol/mL)		4.13 ^a^ ± 0.05	2.95 ^b^ ± 0.05	2.79 ^b^ ± 0.06	0.005

^a,b,c^ Means having different letters in the same row are significantly different (*p* ≤ 0.05). RBCs = red blood cells. Hb = hemoglobin. WBC_S_ = white blood cells. HDL = high-density lipoprotein. LDL = low-density lipoprotein. AST = aspartate aminotransferase. ALT = alanine transaminase. IgA = immunoglobulin A. IgG = immunoglobulin G. IgM = immunoglobulin M. T_3_ = triiodothyronine. TAC = total antioxidant capacity. MDA = malondialdehyde.
